# BHLHE40 confers a pro-survival and pro-metastatic phenotype to breast cancer cells by modulating HBEGF secretion

**DOI:** 10.1186/s13058-018-1046-3

**Published:** 2018-10-01

**Authors:** Aarti Sethuraman, Martin Brown, Raya Krutilina, Zhao-Hui Wu, Tiffany N. Seagroves, Lawrence M. Pfeffer, Meiyun Fan

**Affiliations:** Department of Pathology and Laboratory Medicine, and the Center for Cancer Research, University of Tennessee Health Science Center, 19 South Manassas Street, Memphis, TN 38163 USA

**Keywords:** BHLHE40, Hypoxia, Breast cancer, Metastasis, Exosomes, HBEGF

## Abstract

**Background:**

Metastasis is responsible for a significant number of breast cancer-related deaths. Hypoxia, a primary driving force of cancer metastasis, induces the expression of BHLHE40, a transcription regulator. This study aimed to elucidate the function of BHLHE40 in the metastatic process of breast cancer cells.

**Methods:**

To define the role of BHLHE40 in breast cancer, BHLHE40 expression was knocked down by a lentiviral construct expressing a short hairpin RNA against BHLHE40 or knocked out by the CRISPR/Cas9 editing system. Orthotopic xenograft and experimental metastasis (tail vein injection) mouse models were used to analyze the role of BHLHE40 in lung metastasis of breast cancer. Global gene expression analysis and public database mining were performed to identify signaling pathways regulated by BHLHE40 in breast cancer. The action mechanism of BHLHE40 was examined by chromatin immunoprecipitation (ChIP), co-immunoprecipitation (CoIP), exosome analysis, and cell-based assays for metastatic potential.

**Results:**

BHLHE40 knockdown significantly reduced primary tumor growth and lung metastasis in orthotopic xenograft and experimental metastasis models of breast cancer. Gene expression analysis implicated a role of BHLHE40 in transcriptional activation of heparin-binding epidermal growth factor (HBEGF). ChIP and CoIP assays revealed that BHLHE40 induces HBEGF transcription by blocking DNA binding of histone deacetylases (HDAC)1 and HDAC2. Cell-based assays showed that HBEGF is secreted through exosomes and acts to promote cell survival and migration. Public databases provided evidence linking high expression of BHLHE40 and HBEGF to poor prognosis of triple-negative breast cancer.

**Conclusion:**

This study reveals a novel role of BHLHE40 in promoting tumor cell survival and migration by regulating HBEGF secretion.

## Background

One in every eight women in the USA will be diagnosed with breast cancer over the course of her lifetime [1]. An estimated 266,120 new cases are expected, and 40,920 women are expected to die from breast cancer in 2018 in the USA [1]. Distant metastasis is the major cause of breast cancer-related deaths. Hypoxia has been recognized as a primary driving force of distant metastasis of breast cancer [2–7]. Among hypoxia-responsive genes are both promoting and suppressive factors for malignant progression. It is unclear how the expression and activities of metastasis-promoting factors are preferentially augmented in metastatic tumors. A large body of studies have focused on elucidating the molecular mechanisms by which hypoxia enables cancer cells to survive a variety of stresses imposed by the metastatic process, including nutrient depletion, loss of attachment, and deprivation of growth factors.

Hypoxia-induced exosomic secretion of cytokines and growth factors plays a key role in promoting metastasis through both tumor autonomous and non-autonomous mechanisms [8, 9]. Exosomes are microvesicles (40–130 nm) constitutively released by a variety of cells into the extracellular environment to promote cell-to-cell communication [10]. Tumor cells have been reported to utilize exosomes to transfer nucleotides, lipids, and proteins into surrounding cells or cells in distant metastatic niches [10, 11]. Hypoxia is known to markedly increase the number of secreted exosomes, as well as alter the contents of exosomes [12]. However, our understanding of the regulation of exosome secretion is rudimentary.

Although the cellular response to hypoxia is mainly controlled by two basic helix-loop-helix transcription factors, hypoxia inducible factor (HIF)1A and EPAS1/HIF2A, the outcomes of the hypoxia response are modified by other transcription regulators that are regulated by hypoxia or interact with HIF1A or EPAS1 [13, 14]. This study focuses on the role of a basic helix-loop-helix transcription factor BHLHE40 (also known as DEC1/BHLHB2/SHARP2/STRA13) in metastasis of breast cancer. BHLHE40 expression is directly activated by HIF1A in a variety of tumor cells under hypoxia [15, 16]. High BHLHE40 expression has been linked to activation of a hypoxia-response pathway, elevated metastatic potentials, and poor prognosis of various types of tumors, including hepatocellular carcinoma, pancreatic cancer, and invasive breast cancer [17–19]. It was reported that BHLHE40 binds to the E-box elements and regulates the expression of genes associated with circadian rhythm, cell differentiation, cell senescence, lipid metabolism, DNA damage response, and immune response [20–23]. However, the mechanism of action and downstream targets of BHLHE40 in breast cancer cells is largely unknown. In this study, we provide evidence which suggests that BHLHE40 is a pro-metastasis factor in breast cancer cells which promotes tumor cell survival and migration by modulating exosomic secretion of heparin-binding epidermal growth factor (HBEGF).

## Methods

### Cell culture

Breast cancer MDA-MB-231 and MCF7 cells were obtained from ATCC (Manassas, VA, USA) and maintained in minimal essential medium (ThermoFisher Scientific, Rockford, IL, USA) supplemented with 10% fetal bovine serum (FBS), 200 U/ml penicillin-streptomycin, and 0.5 μg/ml amphotericin B (Cellgro, Manassa, VA, USA). A lung metastatic derivative of MDA-MB-231 (LM) and a tamoxifen-resistant derivative of MCF7 (TR) were established as described previously [24, 25]. A stable line (BHLHE40-KD) expressing a short hairpin RNA (shRNA) against BHLHE40 (TRCN0000232187, Sigma-Aldrich, St. Louis, MO, USA) was generated by lentiviral transduction and selected in medium containing 2 μg/ml puromycin (Sigma-Aldrich). A colony of BHLHE40 knockout variant (BHLHE40-KO) of MDA-MB-231 was generated by using the CRISPR/Cas9 all-in-one expression system (HCP221270-CG01–1, GeneCopoeia) and selected in medium supplemented with 500 μg/ml gentamicin. Elimination of BHLHE40 expression was examined by immunoblotting and quantitative polymerase chain reaction (qPCR) with a forward primer designed to cover the CRISPR editing site (forward 5’GACGGGGAATAAAGCGGAGC and reverse 5’CCGGTCACGTCTCTTTTTCTC). To knockout HIF1A and EPAS1 by CRISPR/Cas9 editing, gRNA targeting exon 1 of HIF1A or EPAS1 was individually cloned into the pX462-puromycin and pX462-hygromycin vectors (expressing Cas9n, AddGene), respectively. MDA-MB-231 cells were transfected with pX462-puro-HIF1A gRNAs using FuGene HD followed by selection in puromycin. A clonal line that had no HIF1A protein detected by immunoblotting was transfected with pX462-hygromycin-EPAS1 gRNA and selected by hygromycin. Hygromycin-resistant colonies that had no EPAS1 detected by immunoblotting were pooled to generate a HIF1A/EPAS1 double-knockout (HIF-dKO) subline. For all knockdown (KD) or KO sublines, control cells were transfected with corresponding empty vectors (EV) and selected in antibiotics in parallel with cells transfected with shRNAs or gRNAs. Pooled drug-resistant colonies of control cells were used as EV control lines. Cells were exposed to different conditions relevant to solid tumors, including loss of attachment (suspension culture), hypoxia (1% O_2_), hypoxia in combination with low (1 mg/ml) glucose (1%O_2_/LG), and hypoxia in combination with glucose depletion (1%O_2_/GF, a condition that induces rapid apoptosis of tumor cells).

### Orthotropic xenograft and experimental lung metastasis models

All in-vivo studies were performed in accordance with the protocols approved by the Institutional Animal Care and Use Committee (IACUC) of the University of Tennessee Health Science Center. NOD.Cg-PrkdcscidIl2rgtm1Wjl/SzJ (NSG) mice were purchased from Jackson Laboratories (Bar Harbor, ME, USA). Orthotropic xenograft and experimental lung metastasis (tail vein injection) models were established using fluorescence-labeled tumor cells, as previously described [24, 26]. Tumor size was monitored and measured weekly using digital calipers. Tumor volume was calculated as: volume = (width^2^ × length)/2. Lung metastasis was quantified by fluorescent imaging of lungs and qPCR of human Alu DNA repeats (forward primer: 5′: GTCAGGAGATCGAGACCATCCC 3′; reverse primer: 5′: TCCTGCCTCAGCCTCCCAAG 3′). Circulating tumor cells in whole blood collected by cardiac puncture were isolated using the Ficoll-Paque PLUS medium (GE Healthcare Life Sciences, Piscataway, NJ, USA) and counted under fluorescent microscope.

### Migration, invasion, and wound healing assays

Transwell membrane inserts with 8-μm pores (BD Biosciences, Bedford, MA, USA), uncoated or coated with Matrigel, were used to determine the migratory and invasive activities of cancer cells, respectively. Cells undergoing migration and invasion were expressed as: percent migration = mean number of cells migrating through the uncoated transwell × 100/mean number of seeded cells; percent invasion = mean number of cells migrating through the Matrigel-coated transwell × 100/mean number of migrating cells through the uncoated pores. Real-time assessment of migratory activity during scratch wound healing was performed using the IncuCyte ZOOM-ImageLock plate system (Essen Bioscience, Michigan, USA). To examine the expression levels of proteins in response to scratch wounds, the EMD Millipore Chemicon Cell Comb Scratch assay kit (Millipore) was used to generate a high-density field of scratches in a confluent cell monolayer to maximize the area of wound edges. To examine the effect of HBEGF on cell migration and invasion, a neutralizing antibody to HBEGF (10 μg/ml; AF-259-NA, R&D systems, Minneapolis, MN, USA) or a HBEGF peptide (20 μg/ml) was added to the medium.

### Suspension culture, viable cell counting, and caspase assays

To mimic the loss of attachment, cells were cultured in PolyHEMA (Sigma-Aldrich)-coated plates to prevent adherence. Methylcellulose (1%) was added to the medium to prevent formation of large cell aggregates to accurately measure tumor cell proliferation in the suspension. Viable cells were counted by the Trypan blue exclusion method. Cell apoptosis was determined using the caspase Glo 3/7 assay kit (Promega, Madison, WI, USA) or immunoblotting of cleaved Caspase 9.

### Luciferase reporter assay for HIF activity

Luciferase reporter constructs driven by hypoxia responsive elements of LDHA (LDHA-Luc, S721613) and ITGA6 (ITGA6-Luc, S708174) were purchased from SwitchGear Genomics (Carlsbad, CA, USA). Cells (3 × 10^5^) were transfected with 500 ng LDHA-Luc or ITGA6-Luc and 10 ng CMV-β-galactosidase (a control for transfection efficiency), along with 250 ng of an empty vector or a HIF1A-expressing construct (HsCD00444875, DNASU plasmid repository), using Fugene6 (Promega). Forty-eight hours after transfection, the cells were exposed to hypoxia (1% O_2_) for 6 h. Luciferase and β-galactosidase (β-gal) activities were measured using the LightSwitch Luciferase Assay System and Promega Beta-Glo Assay System, respectively. Relative luciferase activities normalized to β-gal were presented as mean ± SD, *n* = 6.

### Exosome isolation and analysis

To isolate exosomes, 5 × 10^6^ cells were cultured in medium supplemented with exosome-free serum (SystemBio, Palo Alto, CA, USA). Exosomes in the conditioned medium were purified using the ExoQuick-TC solution (SystemBio) and quantified under a fluorescent microscope after being labeled with carboxyfluorescein diacetate succinimidyl ester (CFSE) using the Exo-Glow labeling kit (SystemBio) that is designed to exclude background particles. To analyze the protein content, isolated exosomes were lysed in RIPA buffer (Pierce, ThermoScientific) containing protease inhibitor cocktail (Sigma-Aldrich) and subjected to immunoblot analysis. To examine the effect of purified exosomes on cell migration, exosomes were re-suspended in exosome-free medium and added to cells seeded in transwells.

### Gene expression microarray and qPCR analysis

Total RNA from cells exposed to hypoxia (1% O_2_ for 6 and 48 h) was purified using the RNeasy kit (Qiagen) and submitted to the Molecular Resource Center at the University of Tennessee Health Science Center for labeling and hybridization to the HT-12 expression BeadChips (Illumina, Chicago, IL, USA). Hybridization signals were processed for annotation, background subtraction, quantile normalization, and presence call filtering using the Gene Expression Module of the Genome Studio Software (Illumina). The microarray data can be found in the Gene Expression Omnibus database with accession number GSE107300. Hypoxia-responsive genes were defined as genes whose expression was altered ≥ 1.5-fold (hypoxia versus control) in two independent experiments. BHLHE40 target genes were defined as genes whose expression was altered ≥ 1.5-fold (BHLHE40-KD versus EV) in two independent experiments. To examine the effect of BHLHE40-KD on gene expression in cells exposed to hypoxia (1% O_2_) in combination with low glucose (1 mM; 1%O_2_/LG), a condition frequently encountered by cells in solid tumors, total RNA of cells exposed to 1%O_2_/LG for 4 h from three independent experiments were pooled and analyzed using the GeneChip Human Gene 1.0 ST array (Affymetrix, Santa Clara, CA, USA). The Affymetrix data were extracted, normalized, and summarized with the robust multi-average (RMA) method implemented in the Affymetrix Expression Console. To validate microarray data by qPCR, total RNA from cells in three independent experiments with duplicates was prepared using trizol (Life Technologies, Grand Island, NY, USA). cDNAs were synthesized using iScript cDNA Synthesis kits (Bio-Rad, Hercules, CA, USA) and qPCR was performed on the CFX96TM Real-Time PCR detection system using SYBR green supermix (Bio-Rad). Expression data of mRNA were normalized by the 2^–ΔΔCT^ method to RPL13A and presented as mean ± SD. Primers for qPCR were obtained from the Primerbank [27].

### Protein extraction, co-immunoprecipitation, and immunoblotting

For immunoblotting (IB) analysis, whole cell lysates and nuclear proteins were prepared using RIPA buffer supplemented with protease inhibitor cocktails (Sigma-Aldrich) and the NE-PER Nuclear and Cytoplasmic Extraction Reagents (Thermo Scientific), respectively. To detect protein-protein interaction, soluble proteins were extracted using the Pierce IP Lysis Buffer (Thermo Scientific) supplemented with protease inhibitor cocktails and co-immunoprecipitation (CoIP) was performed using the TrueBlot Immunoprecipitation and Western Blot Kit (Rockland Immunochemicals Inc., Limerick, PA, USA). IB signals were developed using the SuperSignal West Dura Extended Duration Substrate and the CL-XPosure Film (Thermo Scientific). Antibodies used in this study were: anti-EGFR-Tyr1110P, anti-EGFR, anti-ERK1/2-Thr202/Tyr204P, anti-ERK1/2, anti-AKT-Tyr416P, anti-AKT, anti-Caspase9, anti-HDAC1, and anti-HDAC2 from Cell Signaling Technologies (Boston, MA, USA), anti-GAPDH from Millipore (Merck, Darmstadt, Germany), anti-TBP from Abcam (Cambridge, MA, USA), anti-CTGF from Abgent (San Diego, CA, USA), anti-HBEGF and anti-CD9 from R&D Biosystems (Minneapolis, MN, USA), anti-BHLHE40, anti-HIF1A, and anti-EPAS1 from Novus Biologicals (Littleton, CO, USA), and anti-ALIX, anti-TSG101, and anti-CD81 from Santa Cruz (Dallas, TX, USA).

### Chromatin immunoprecipitation (ChIP)

Protein-DNA crosslink, nuclear fraction extraction, and chromatin fragmentation were performed as described previously [26]. The soluble fraction of sheared chromatin (200–500 bp in length) was pre-cleaned with Magnabind Goat anti-Rabbit IgG (for anti-BHLHE40 and anti-HDAC1) or Magnabind Goat anti-Mouse (for anti-HDAC2) (Life Technologies), followed by immunoprecipitation with control IgG or antibodies against BHLHE40, histone deacetylases (HDAC)1 or HDAC2, and Magnabind beads conjugated with a secondary antibody. DNA in the de-crosslinked immunocomplexes was isolated with the MiniElute PCR purification kit (Qiagen, Germantown, MD, USA). qPCR was performed to detect the presence of the proximal promoter region of HBEGF (−529 to −372 from the transcription start site) using the following primers: forward 5’TGCCTGCAACTTCAACT CTG3′ and reverse 5’CCATCCCTGTCACCCTCTAA3′.

### Statistical analysis

Student’s *t* tests, one-way analysis of variance (ANOVA) with post-hoc Tukey test and correlation significance analyses were performed using the GraphPad Prism 5 software (GraphPad, San Diego, CA, USA); *p* values < 0.05 were considered statistically significant.

## Results

### BHLHE40 knockdown leads to decreased primary tumor growth and lung metastases

**Fig. 1:**
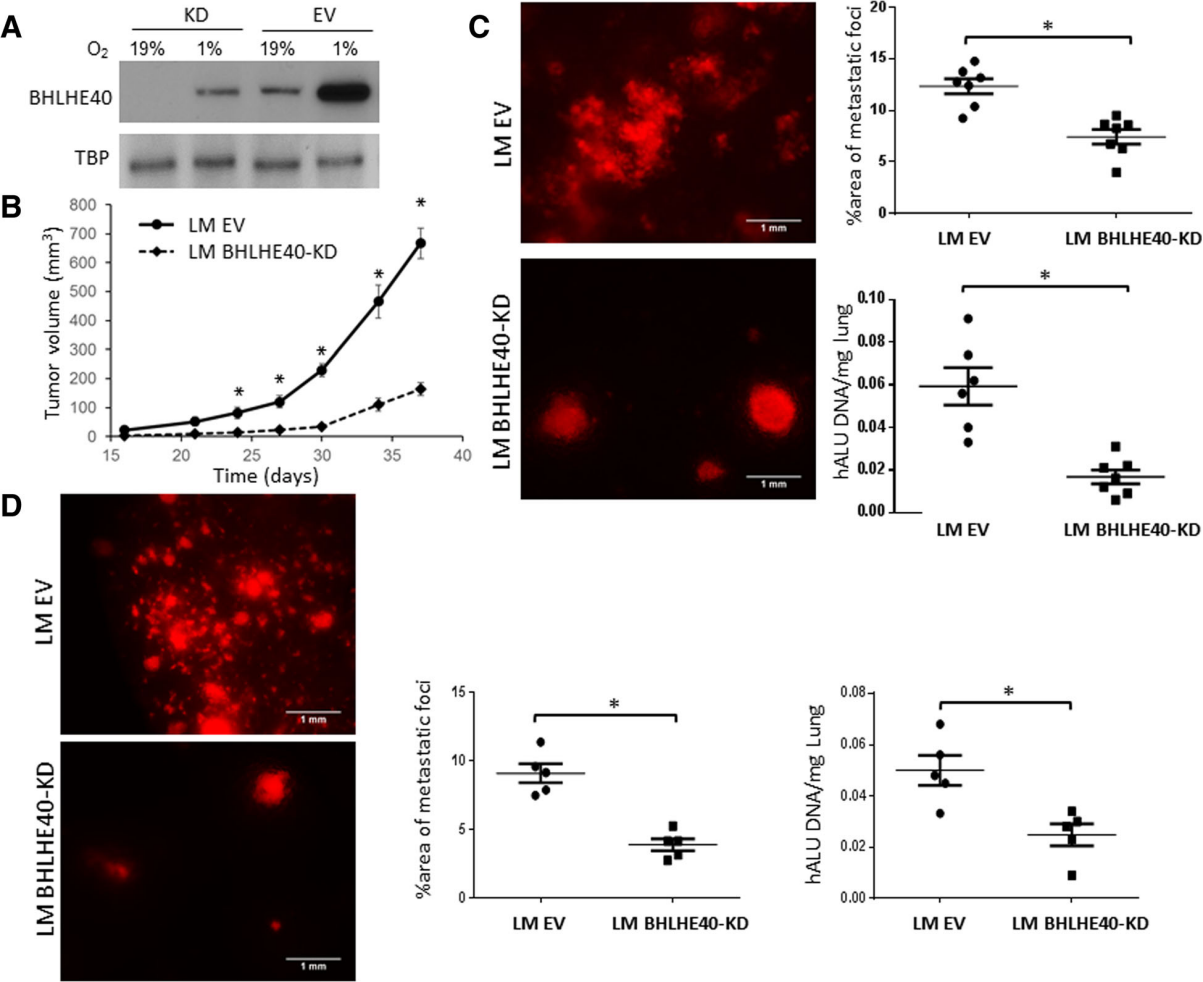
BHLHE40-knockdown (KD) significantly reduced primary tumor size and lung metastatic burden in an orthotopic xenograft model. **a** BHLHE40-shRNA expression effectively reduced both baseline and hypoxia-induced expression of BHLHE40 protein in the LM cells, as determined by immunoblotting. **b** Orthotopic xenograft tumors derived from LM-BHLHE40-KD cells exhibited lower growth rate than tumors derived from control LM empty vector (EV) cells. NSG mice were inoculated in the inguinal mammary gland fat pads with 2 × 10^5^ cells. Tumor size was monitored and measured weekly using a digital caliper. Tumor volume was calculated as: volume = (width^2^ × length)/2. *p <  0.05 (*n* = 20, KD vs. EV at indicated time points), one-way ANOVA followed by Tukey’s post-hoc tests. **c** Spontaneous lung metastasis detected by fluorescent imaging of lungs or human ALU repeats qPCR 5 weeks after inoculation of tumor cells in mammary gland fat pads. *p <  0.05 (*n* = 10, KD vs. EV), Student’s *t* test. **d** Lung metastasis in mice after resection of primary tumors. Primary tumors in mammary gland fat pads were resected when they reached a size of 5 × 5 mm and lung metastasis were analyzed 4 weeks post-resection by fluorescent imaging of lungs or human ALU repeats qPCR. *p <  0.05 (*n* = 10, KD vs. EV), Student’s *t* test

To define the role of BHLHE40 in breast cancer metastasis, we examined the effect of its knockdown (KD) by a shRNA lentiviral construct on spontaneous lung metastasis of orthotopic xenograft tumors derived from a lung metastasis-enriched subline (LM) of breast cancer MDA-MB-231 cells [28]. The protein levels of BHLHE40 is low in cells under normal growth conditions but is significantly induced by hypoxia (1% O_2_, 16 h). BHLHE40-shRNA expression effectively reduced both baseline and hypoxia-induced levels of BHLHE40 in LM cells (Fig. 1a). In NSG mice inoculated with 2 × 10^5^ control LM-EV (empty vector) cells in the inguinal mammary gland fat pads, palpable tumors were detected at 2 weeks (Fig. 1b) and lung metastasis became evident at 5 weeks (Fig. 1c) post-inoculation. BHLHE40-KD delayed the onset of primary tumors, which became palpable 3 weeks after inoculation, and reduced the growth rate of primary tumors, coincident with decreased lung metastases (Fig. 1a–c). To further investigate the effect of BHLHE40-KD on lung metastases, primary tumors of EV and BHLHE40-KD cells were surgically removed at 3 and 5 weeks post-inoculation, respectively, when they reached similar size with a diameter of 4–5 mm. Lung metastasis was examined 4 weeks after primary tumor resection (Fig. 1d). BHLHE40-KD substantially reduced lung metastasis in mice with similar primary tumor burdens. Taken together, these results suggest that BHLHE40 plays a role in promoting primary tumor growth and spontaneous distant metastasis of breast cancer cells.

### BHLHE40 knockdown reduces lung colonization of tumor cells inoculated through tail vein

**Fig. 2:**
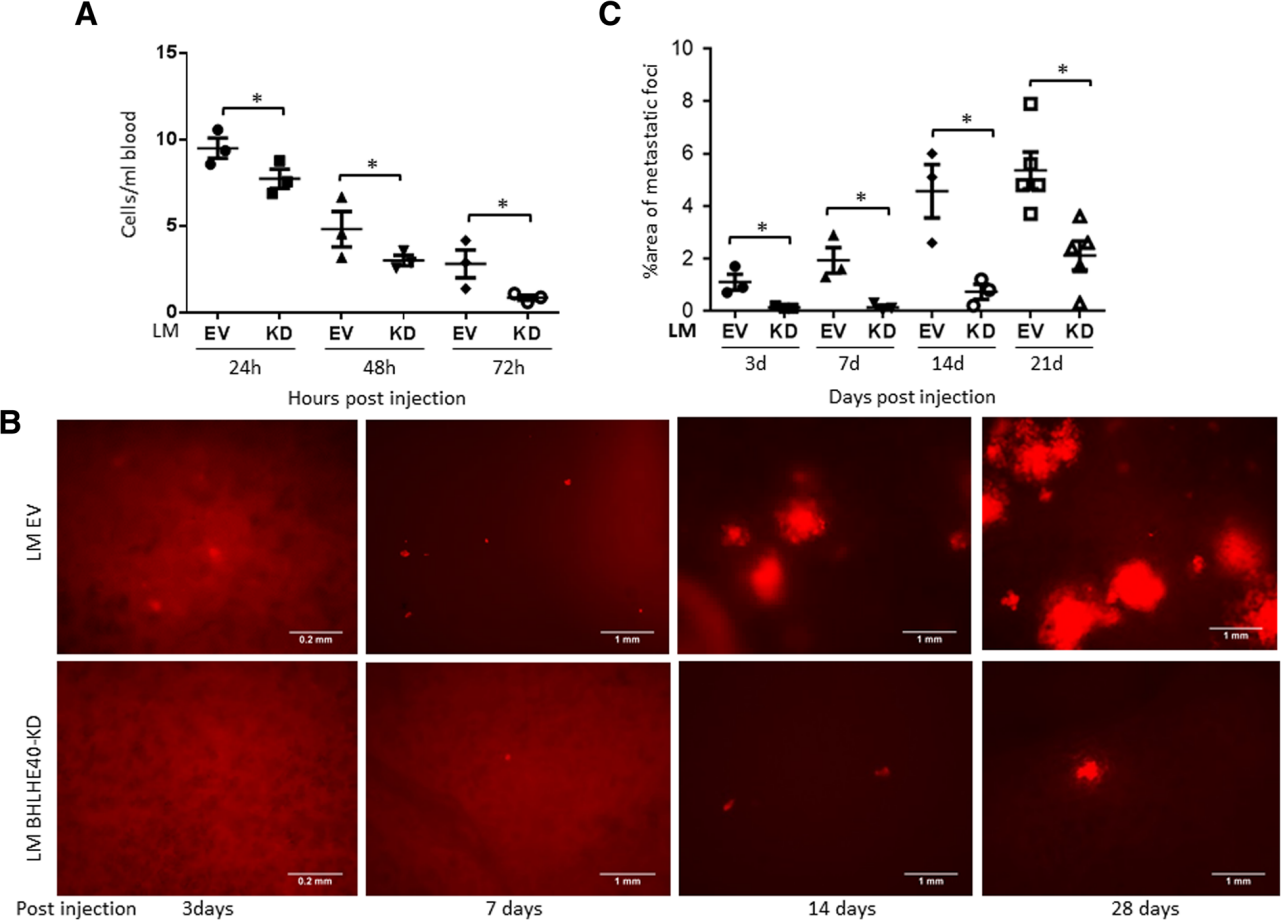
BHLHE40-knockdown (KD) reduced lung colonization of tumor cells inoculated into the circulation via tail veins. **a** Less tumor cells were detected in blood of NSG mice inoculated via tail vein injection with LM BHLHE40-KD than mice inoculated with empty vector (EV) control LM EV cells. Tumor cells in whole blood collected by cardiac puncture at indicated times after tail vein injection were isolated using the Ficoll-Paque PLUS medium and counted under fluorescent microscope. *p <  0.05 (*n* = 3, KD vs. EV), one-way ANOVA followed by Tukey’s post-hoc tests. **b** Fluorescent imaging of metastatic foci in lungs at different time points post tail-vein injection. **c** Percentage of areas occupied by metastatic loci in the lungs, as quantified by fluorescent imaging and ImageJ. *p <  0.05 (*n* = 3, KD vs. EV), one-way ANOVA followed by Tukey’s post-hoc tests

To determine whether BHLHE40 regulates late metastatic events after entry of tumor cells into the blood stream, we examined the effect of BHLHE40-KD on the ability of tumor cells to survive circulation and colonize in the lungs using an experimental metastasis model, in which tumor cells were delivered into the blood stream through tail vain injection to bypass the initial steps of metastasis such as migration and intravasation. LM-EV and LM-BHLHE40-KD cells (5 × 10^5^) were injected into the left lateral tail veins of 5-week-old female NSG mice, and tumor cells in the bloodstream and lung tissues were examined at various times post-injection (Fig. 2). Compared with control LM-EV cells, LM-BHLHE40-KD cells were more rapidly eliminated from the bloodstream (Fig. 2a). LM-EV cells were observed in lung tissues at 72 h and formed large metastatic foci at 4 weeks after tail vein injection (Fig. 2b, c). In contrast, BHLHE40-KD cells were not detected in lung tissue at 72 h and formed less metastatic foci in lungs than EV cells at various time points (Fig. 2b, c). No fluorescent loci of EV or BHLHE40-KD cells were found in other organs (i.e., livers, spleens, and kidneys) within 5 weeks after tail vein inoculation. Together, these results suggest that BHLHE40 is required for tumor cells to survive in the circulation and establish metastatic foci in the lungs.

### BHLHE40 acts to promote cell migration, invasion and survival

**Fig. 3:**
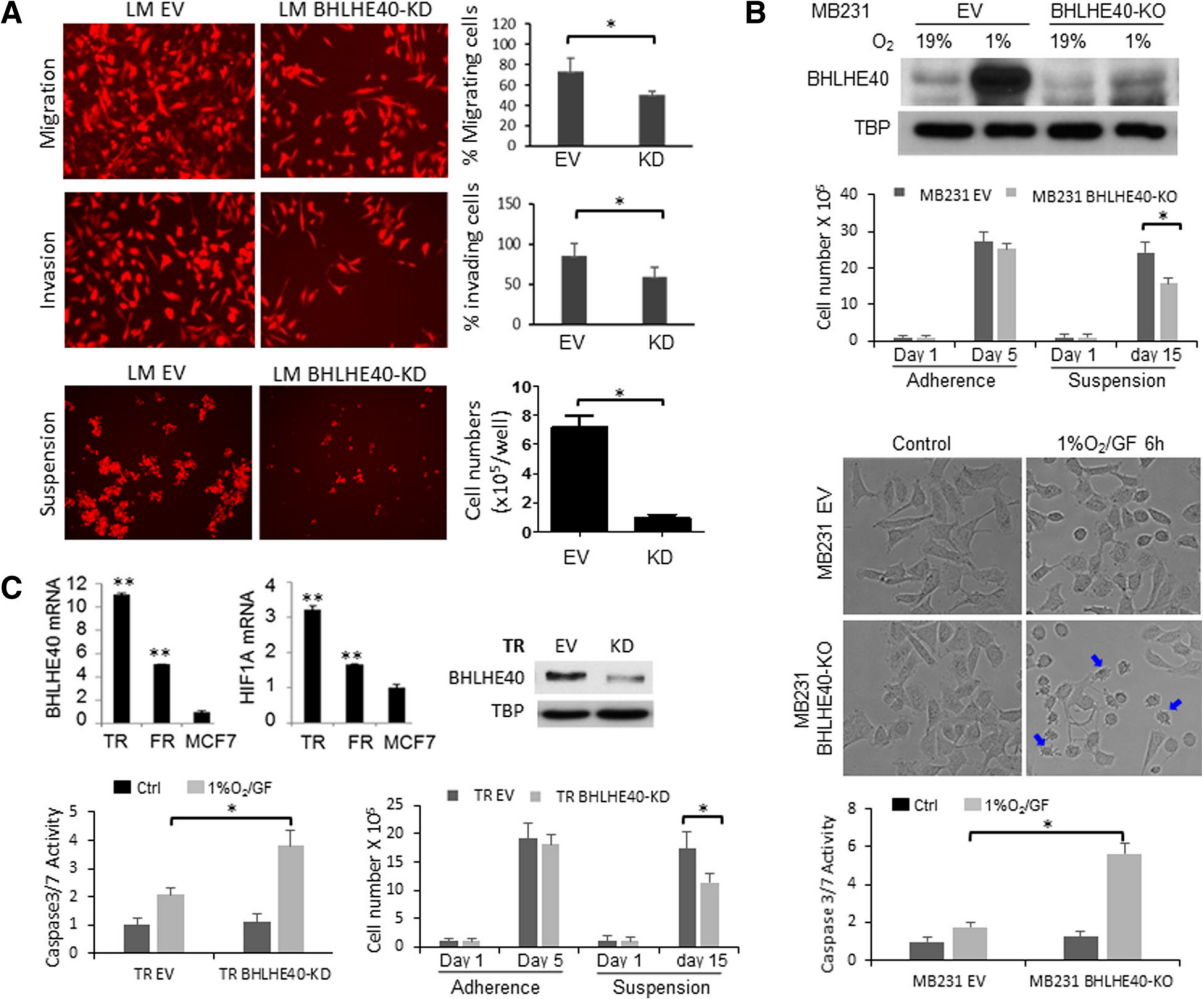
BHLHE40 depletion impaired cell migration, invasion, and survival. **a** BHLHE40-knockdown (KD) by shRNA in LM cells reduced cell migration and invasion, as well as the number of viable cells during suspension culture in comparison to empty vector (EV) control LM EV cells. Migratory and invasive activities were determined using transwells, uncoated or coated with Matrigel, respectively. The results were presented as: percent migration = mean number of cells migrating through the uncoated transwells × 100/mean number of seeded cells; percent invasion = mean number of cells migrating through the Matrigel-coated transwells × 100/mean number of migrating cells through the uncoated transwells. Suspension culture was conducted by seeding cells in medium containing 1% methylcellulose and in dishes coated with PolyHEMA for 15 days. Viable cells were counted under fluorescent microscope. *p <  0.05 (*n* = 6, KD vs. EV), Student’s *t* test. **b** BHLHE40-knockout (KO) by CRISPR/Cas9 editing in MDA-MB-231 cells reduced the number of viable cells after suspension culture and enhanced apoptosis induced by hypoxia combined with glucose depletion (1%O_2_/GF). Viable cells were determined by Trypan blue exclusion-based cell counting after a 15-day suspension culture. Apoptosis of cells exposed to 1%O_2_/GF for 6 h was examined by the appearance of apoptotic morphology (as indicated by arrows in the cell images) and Caspase3/7 assays. *p <  0.05 (*n* = 6, KO vs. EV), one-way ANOVA followed by Tukey’s post-hoc tests. **c** BHLHE40-KD by shRNA in tamoxifen-resistant subline of MCF7 (TR) reduced the ability of cells to survive 1%O_2_/GF (6 h) and reduced the number of viable cells after a 15-day suspension culture. Elevated expression of HIF1A and BHLHE40 in TR and fulvestrant-resistant (FR) sublines of MCF7 cells were detected by qPCR. BHLHE40-KD was confirmed by immunoblotting. Apoptosis induced by 1%O_2_/GF (6 h) was determined by Caspase3/7 assays. The number of viable cells after a 15-day suspension culture was determined by Trypan blue exclusion-based cell counting. **p <  0.05 (*n* = 6, TR or FR vs. parent MCF7), *p <  0.05 (*n* = 6, KD vs. EV), one-way ANOVA followed by Tukey’s post-hoc tests

Having established a role for BHLHE40 in distant metastasis of breast cancer cells in vivo, we sought to identify the specific cellular processes that require BHLHE40 activity. Despite the significant effect of BHLHE40-KD on primary tumor growth and lung metastasis of LM cells in vivo, BHLHE40-KD showed no significant effect on proliferation of LM cells under normal two-dimensional growth conditions in vitro. The doubling times, determined by Trypan blue exclusion-based cell counting (daily for 7 days), of LM-EV and LM-BHLHE40-KD cells were 36.37 ± 0.49 h (*n* = 6) and 38.95 ± 3.61 h (n = 6), respectively. Therefore, we focused on investigating whether BHLHE40 is a downstream effector of HIF1A activation by hypoxia or loss of attachment. Detached breast cells were reported to rely on HIF1A activation to survive under normoxia [29]. In-vitro cell migration and invasion assays showed that BHLHE40-KD reduced the ability of cells to penetrate either uncoated or Matrigel-coated transwells under hypoxia conditions (1% O_2_; Fig. 3a). Under the nonadherent culture condition for 15 days, in which cells were mixed with growth medium supplemented with 1% methylcellulose to prevent cell aggregation and then seeded in plates coated with polyHEMA to prevent adherence, the number of viable LM BHLHE40-KD cells was significantly lower than LM EV cells (Fig. 3a, lower panel). To examine whether the effects exerted by BHLHE40-KD on LM cells can be extended to the parent MDA-MB-231 cells, we established a BHLHE40 knockout (KO) subline using the CRISPR/Cas9 editing system. BHLHE40 protein depletion in the KO subline under normoxia or hypoxia was confirmed by immunoblotting (Fig. 3b, upper panel). Although residue BHLHE40 protein was detected in the KO subline by immunoblotting, no wild-type mRNA was detected by qPCR with a forward primer designed to cover the CRISPR editing site. BHLHE40-KO resulted in a reduced number of viable cells after a 15-day suspension culture in plates coated with polyHEMA to prevent attachment (Fig. 3b, middle panel), while it showed no significant effect on the number of viable cells after a 5-day adherence culture. In addition, BHLHE40-KO significantly sensitized MDA-MB-231 cells to apoptosis induced by hypoxia in combination with glucose depletion (1%O_2_/GF, 6 h), as evidenced by the appearance of apoptotic morphology and activation of caspase3/7 (Fig. 3b, lower panel).

We further examined the function of BHLHE40 in breast tumor cells with elevated baseline activation of HIF1A and BHLHE40 using the tamoxifen-resistant (TR) and fulvestrant-resistant (FR) variants of MCF7 cells [25]. As shown in Fig. 3c, mRNA expression levels of BHLHE40 and HIF1A are significantly elevated in TR and FR cells in comparison with parent MCF7 cells. BHLHE40-KD in TR cells substantially increased apoptosis induced by glucose depletion, under both normoxia and hypoxia conditions, as well as reducing the number of viable cells after a 15-day suspension culture in polyHEMA-coated plates (Fig. 3c). Similarly, BHLHE40-KD reduced the number of viable cells in suspension culture and the ability of FR cells to survive glucose depletion (data not shown). Collectively, these observations provide evidence supporting a role for BHLHE40 in promoting survival and migration.

### BHLHE40 is required for transcription activation of a set of cytokines and growth factors

**Fig. 4:**
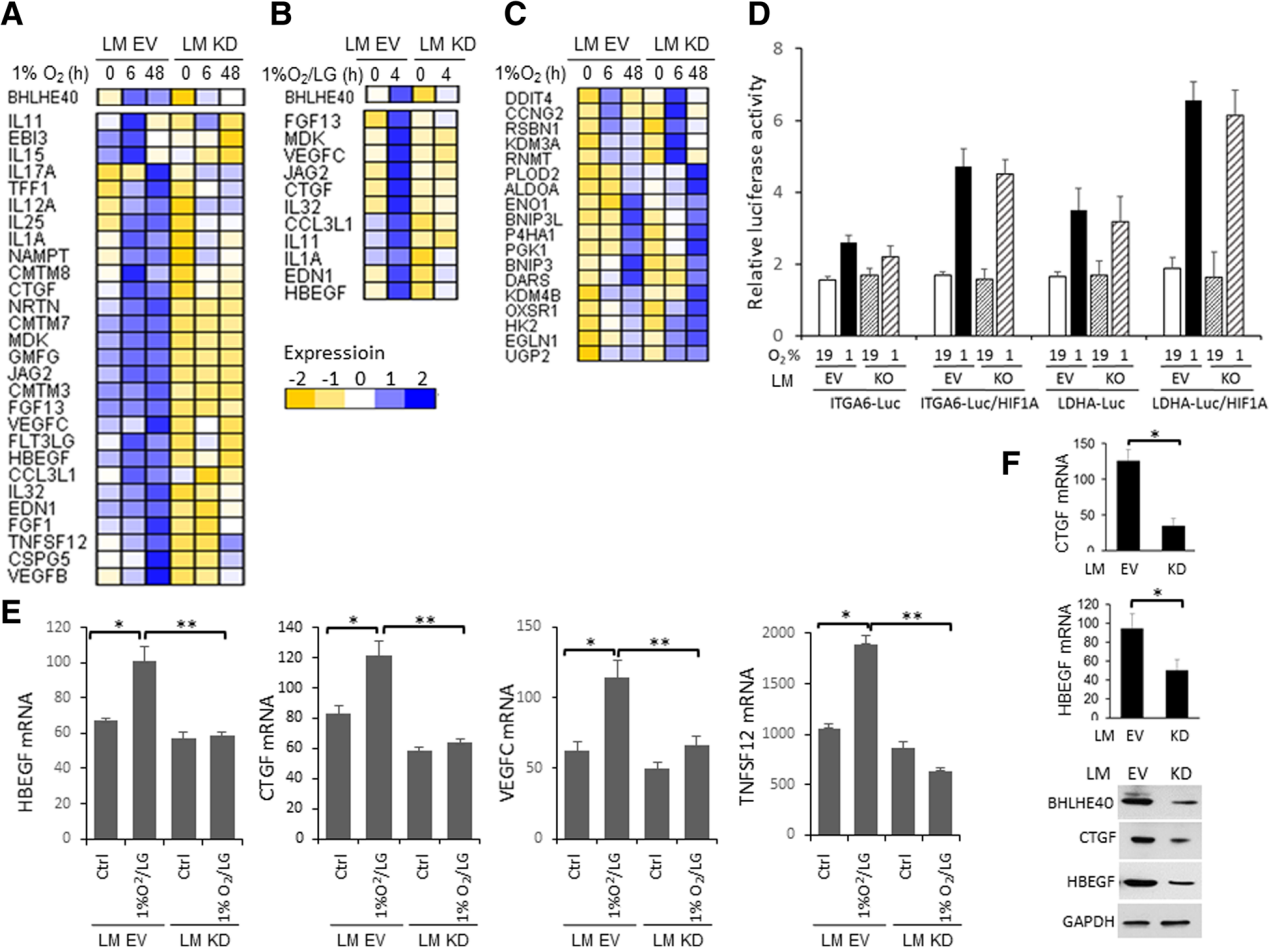
BHLHE40-knockdown reduced hypoxia-induced expression of a panel of cytokines and growth factors. **a** Heatmaps of cytokines and growth factors whose hypoxia-induced expression (1% O_2_ at 6 h or 48 h, fold-change ≥ 1.5 in two independent experiments) was diminished by BHLHE40-knockdown (KD) in LM cells. The gene expression levels were determined using the Illumina Human HT-12 expression BeadChips. Normalized (quantile normalization) hybridization signals were log2 transformed and standardized by genes across experiment conditions to generate the heatmap. **b** Heatmaps of a subset of genes list in **a** whose expression was affected by BHLHE40-KD in LM cells exposed to hypoxia combined with low (1 mM) glucose (1%O_2_/LG, 4 h). The gene expression levels were determined using the Affymetrix Human Gene 1.0 ST array. **c** Heatmaps of hypoxia-induced genes whose expression was not significantly affected by BHLHE40-KD in LM cells as determined by the Illumina Human HT-12 expression BeadChips. **d** Expression of luciferase reporters driven by hypoxia-responsive elements of ITGA6 or LDHA was not affected by BHLHE40 knockout (KO) by CRISPR/Cas9 editing in MDA-MB-231 cells, in the absence or presence of exogenous HIF1A. Luciferase activities were normalized to co-transfected CMV-β-galactosidase and presented as mean ± SD (*n* = 6). **e** Expression of genes in control LM empty vector (EV) and LM BHLHE40-KD cells exposed to 1%O_2_/LG (4 h). mRNA expression levels were determined by qPCR, normalized to RPL13A, and presented as mean ± SD (*n* = 6). *p <  0.05 (*n* = 6, 1%O_2_/LG vs. untreated control), **p <  0.05 (*n* = 6, KD vs. EV), one-way ANOVA followed by Tukey’s post-hoc tests. **f** mRNA and protein expression levels of HBEGF and CTGF in primary xenograft tumors, determined by qPCR and immunoblotting, respectively. *p <  0.05 (*n* = 6, KD vs. EV), Student’s *t* test. Representative immunoblotting images of three tumors of KD or EV cells are presented

To delineate the molecular pathways regulated by BHLHE40, we performed global gene expression analysis of LM-EV and LM BHLHE40-KD cells exposed to hypoxia (1% O_2_, 6 h or 48 h). The microarray data can be found in the Gene Expression Omnibus database with accession number GSE107300. Overall, the expression levels of 521 and 646 genes in LM-EV cells were altered (fold-change ≥ 1.5 in two independent experiments) by hypoxia at 6 h and 48 h, respectively. BHLHE40-KD abolished the hypoxia-mediated upregulation of 45 (out of 261, 17.2%) and 98 (out of 361, 27.1%) genes at 6 h and 48 h, respectively. In addition, BHLHE40-KD abolished the hypoxia-mediated downregulation of 30 (out of 260, 10.5%) and 44 (out of 285, 15.4%) genes at 6 h and 48 h, respectively. The hypoxia-induced genes that were affected by BHLHE40-KD were over-represented by genes that encode proteins with cytokine or growth factor activities as defined by Gene Ontology annotation GO:0005125 and GO:0008083 (Fisher’s exact test, *p* <  0.0001; Fig. 4a). The expression of a subset of these genes was also reduced in BHLHE40-KD cells exposed to 1%O_2_/LG for 4 h compared with EV cell (Fig. 4b). In contrast, hypoxia-induced expression of a panel of the core hypoxia-responsive genes that are known to be directly targeted by HIF1A [14] was not significantly affected by BHLHE40-KD (Fig. 4c). These observations suggest that BHLHE40-KD preferentially reduced the hypoxia-induced expression of a set of cytokines and growth factors but did not cause a global defect in HIF1A-mediated transcription activation. To confirm this notion, we examined the effect of BHLHE40-KO on HIF-mediated expression of reporter luciferase driven by well-characterized HIF1A-binding sites in the promoter regions of LDHA and ITGA6 [26–28]. As shown in Fig. 4d, BHLHE40-KD exhibited no significant effect on hypoxia-induced luciferase activities, in the absence or presence of exogenous HIF1A protein. The effect of BHLHE40-KD on the expression of cytokines and growth factors in cells exposed to 1%O_2_/LG (4 h) was validated by qPCR (Fig. 4e). Consistent with results in BHLHE40-KD cells cultured in vitro, we detected reduced expression of CTGF and HBEGF, at both the mRNA and protein levels, in the LM-BHLHE40-KD primary tumors in comparison with LM-EV tumors established in mouse mammary gland fat pads (Fig. 4f).

To determine whether BHLHE40-mediated expression of genes encoding cytokine or growth factors is relevant to clinical samples, we analyzed the mRNA expression data of breast tumors in The Cancer Genome Atlas (TCGA) database [30, 31]. The expression of 71.4% (20 out of 28) of these BHLHE40-dependent genes (as shown in Fig. 4a) was found to be positively correlated with BHLHE40 expression, with statistical significance (*p* <  0.05) in at least one of the four major subtypes of breast tumors (Table 1). This observation provides supporting evidence of a role for BHLHE40 in the expression of these genes in human breast tumors.

**Table 1:** Correlated expression of BHLHE40 and its putative targets in breast tumors (The Cancer Genome Atlas)

Gene	BL (*n* = 230)		Her2 (*n* = 162)		LA (*n* = 315)		LB (*n* = 300)	
	Pearson *r*	*p* value	Pearson *r*	*p* value	Pearson *r*	*p* value	Pearson *r*	*p* value
CCL3L1	0.1210	0.0694	−0.2045	0.0095	0.0504	0.3792	0.0480	0.4136
CMTM3	−0.0042	0.9494	0.0358	0.6510	0.5065	< 0.0001	−0.0521	0.3687
CMTM7	−0.2761	< 0.0001	−0.0697	0.3780	0.2516	< 0.0001	0.0635	0.2729
CMTM8	−0.1580	0.0165	−0.0886	0.2624	0.1034	0.0670	−0.0013	0.9817
CSPG5	−0.1333	0.0435	−0.0309	0.6961	−0.0351	0.5345	−0.0504	0.3850
CTGF	0.0203	0.7593	0.3087	< 0.0001	0.4876	< 0.0001	0.0631	0.2760
EBI3	0.2135	0.0011	−0.2060	0.0085	0.0805	0.1543	−0.0271	0.6402
EDN1	0.0705	0.2867	0.0928	0.2401	0.3805	< 0.0001	0.0273	0.6379
FGF1	0.1666	0.0114	0.2470	0.0015	0.4225	< 0.0001	0.0357	0.5384
FGF13	−0.1105	0.0946	−0.1782	0.0233	0.1393	0.0134	−0.1276	0.0271
FLT3LG	0.2436	0.0002	−0.0588	0.4573	0.2878	< 0.0001	−0.0264	0.6487
GMFG	0.2593	< 0.0001	−0.1764	0.0247	0.3367	< 0.0001	−0.1271	0.0277
HBEGF	0.3871	< 0.0001	0.2873	0.0002	0.3254	< 0.0001	0.1577	0.0062
IL11	0.1413	0.0322	0.1762	0.0249	0.2053	0.0002	0.0328	0.5710
IL12A	0.0325	0.6207	−0.0582	0.4618	−0.1035	0.0649	−0.1543	0.0069
IL15	0.3407	< 0.0001	0.0628	0.4273	0.1632	0.0037	−0.0913	0.1146
IL17A	0.1036	0.1139	0.0845	0.2850	−0.0221	0.6942	−0.0919	0.1094
IL1A	0.2140	0.0013	−0.0057	0.9440	0.0399	0.4960	−0.0720	0.2359
IL25	−0.1011	0.3633	0.0560	0.6578	−0.1569	0.0293	0.1617	0.0661
IL32	0.1597	0.0153	−0.0571	0.4704	0.3242	< 0.0001	−0.0473	0.4141
JAG2	−0.0418	0.5287	0.0387	0.6250	0.0880	0.1190	−0.0167	0.7729
MDK	−0.0837	0.2057	−0.0947	0.2305	0.1107	0.0496	0.0066	0.9099
NAMPT	0.2678	< 0.0001	0.0873	0.2691	0.1667	0.0030	−0.1819	0.0016
NRTN	−0.4457	< 0.0001	−0.0350	0.6596	0.0672	0.2411	−0.0288	0.6220
TFF1	0.2156	0.0009	−0.0148	0.8516	0.0221	0.6936	−0.1243	0.0300
TNFSF12	0.1690	0.0102	−0.0271	0.7323	0.1482	0.0084	0.0565	0.3296
VEGFB	−0.0460	0.4879	0.0811	0.3049	0.2028	0.0003	0.0443	0.4449
VEGFC	0.3664	< 0.0001	0.1760	0.0251	1.0000	< 0.0001	−0.0922	0.1110

The correlation analysis was performed using the mRNA expression *z* scores (RNA Seq V2 RSEM, The Cancer Genome Atlas)

*BL* basal-like, *Her2* ERBB2-enriched, *LA* luminal A, *LB* luminal B

**Fig. 5:**
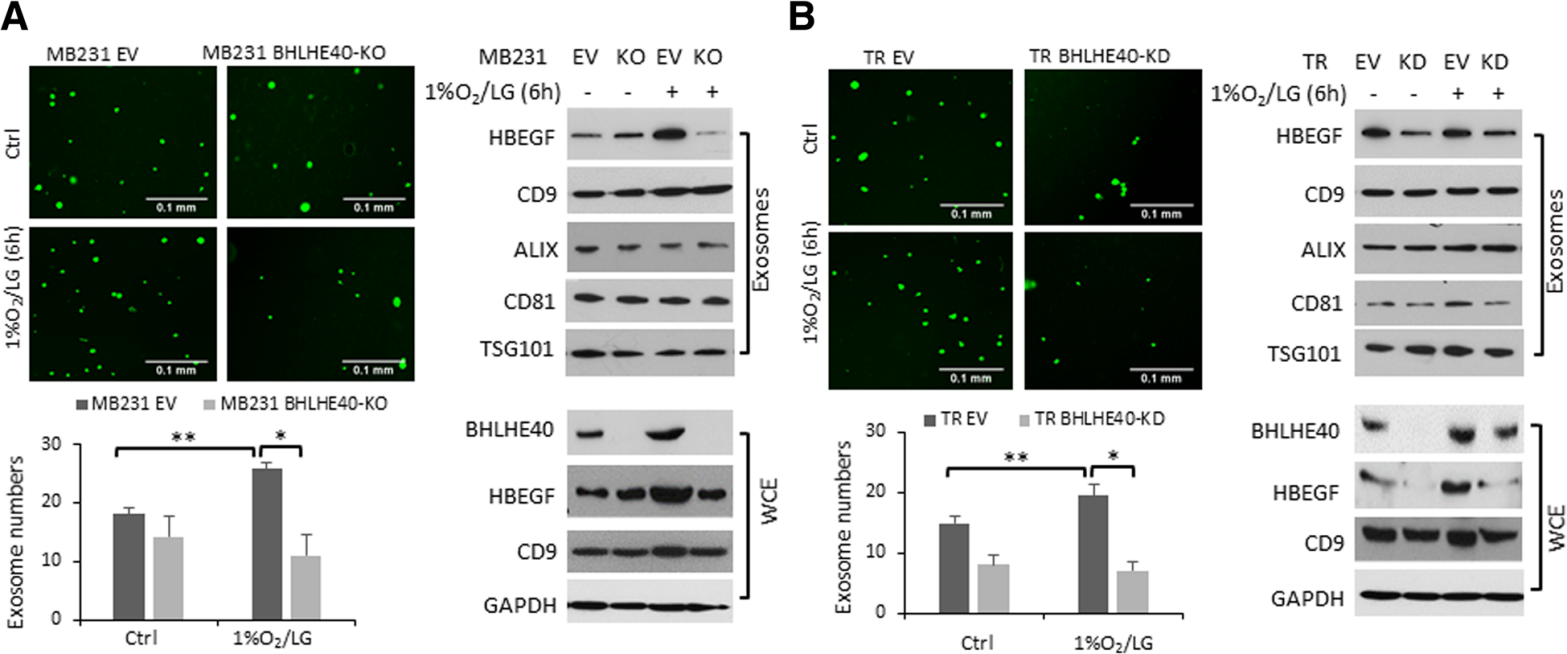
Exosomic secretion of HBEGF was reduced by BHLHE40 depletion in MDA-MB231 or TR cells. **a** BHLHE40-knockout (KO) by CRISPR/Cas9 editing in MDA-MB-231 cells reduced the total number of exosomes and the amount of HBEGF protein in exosomes in comparison with empty vector (EV) control cells. **b** BHLHE40-knockdown (KD) by shRNA in tamoxifen-resistant (TR) cells reduced the total number of exosomes and the amount of HBEGF protein in exosomes in comparison with TR EV cells. Exosomes were purified from conditioned medium of 5 × 10^6^ cells cultured in medium supplemented with exosome-free serum, either under normal culture condition or exposed to 1% O_2_/low glucose (LG) for 6 h. Exosomes were purified using the ExoQuick-TC solution and quantified under a fluorescent microscope after being labeled with carboxyfluorescein diacetate succinimidyl ester (CFSE) using the Exo-Glow labeling kit, which is designed to exclude background particles. Exosome number in the bar graph is presented as mean number of exosomes per field ± SD (total of nine fields from three independent experiments were examined). The presence of HBEGF and exosome markers in purified exosomes (3 μg protein/lane) or whole cell extracts (WCE; 30 μg protein/lane) were detected by immunoblotting. **p <  0.05 (*n* = 9, 1%O_2_/LG vs. control), *p <  0.05 (*n* = 9, KO or KD vs. EV), one-way ANOVA followed by Tukey’s post-hoc tests

Since hypoxia-induced cytokines and growth factors are commonly exported to the extracellular space by exosomes [32], we sought to determine whether BHLHE40-KD could affect exosome secretion. As shown in Fig. 5, the number of isolated exosomes was significantly reduced in the conditioned medium from MDA-MB-231 BHLHE40-KO and TR-BHLHE40-KD cells in comparison with the corresponding control cells cultured under both normal conditions or exposed to 1%O_2_/LG for 6 h. The presence of exosomic markers (i.e., CD9, CD81, ALIX, and TSG101) [33, 34]) in the isolated exosomes was confirmed by immunoblotting (Fig. 5). BHLHE40 depletion reduced the protein levels of HBEGF in the purified exosomes (Fig. 5), reflecting the reduced levels of HBEGF mRNA and HBEGF protein in whole cell extracts of BHLHE40-KD or KO cells (Figs. 4e and 5). These observations suggest that BHLHE40 depletion reduced overall exosome secretion and sorting of HBEGF into exosomes.

### BHLHE40 activates HBEGF transcription by sequestering HDAC1 and HDAC2 from promoter binding

**Fig. 6:**
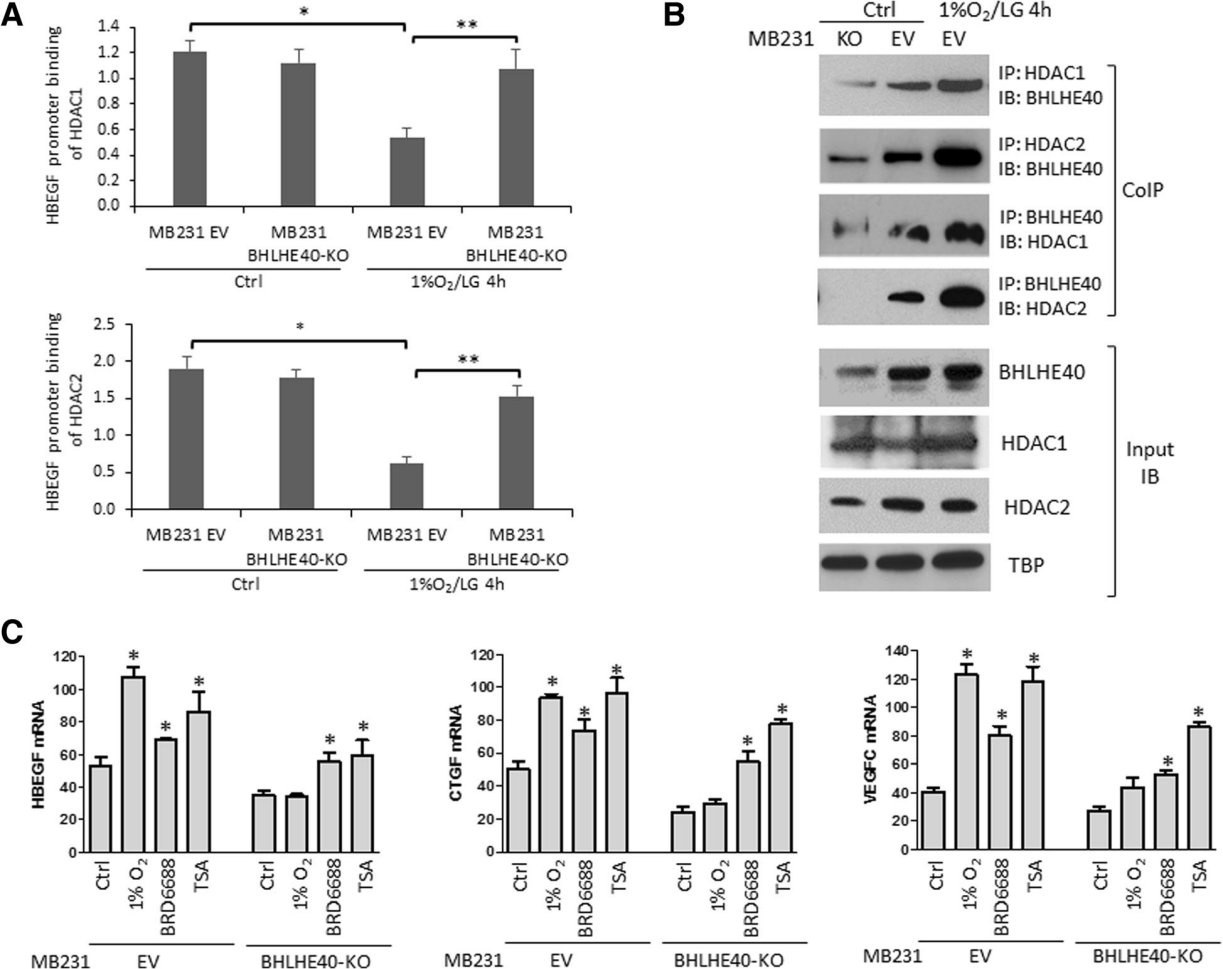
BHLHE40 activates gene expression by sequestering histone deacetylase (HDAC)1 and HDAC2 from genome DNA binding in MDA-MB-231 cells exposed to hypoxia and low glucose (1%O_2_/LG, 4 h). **a** BHLHE40-knockout (KO) diminished dissociation of HDAC1 and HDAC2 from the promoter region of HBEGF in MDA-MB-231 cells exposed to 1%O_2_/LG (4 h), as determined by chromatin immunoprecipitation (ChIP) followed by qPCR of the HBEGF promoter region (−529 to −372 from the transcription start site). HBEGF promoter binding activity of HDAC1 or HDAC2 was calculated as: (DNA amount in anti-HADC IP complex – DNA amount in control IgG IP complex)/DNA amount in 1% input. *p <  0.05 (*n* = 6, 1%O_2_/LG vs. control), **p <  0.05 (*n* = 6, KO vs. EV). **b** 1%O_2_/LG treatment increased interactions between BHLHE40 and HDAC1/2 in the soluble cellular fraction of MDA-MB-231 empty vector (EV) cells. Protein-protein interaction was detected by reciprocal co-immunoprecipitation (IP)/immunoblotting (IB) analysis. **c** HDAC inhibition induced expression of BHLHE40 target genes. Cells were exposed to hypoxia (1% O_2_) or HDAC inhibitors (BRD6688 10 μM or TSA 2 μM) for 24 h. mRNA expression levels were determined by qPCR, normalized to RPL13A, and presented as mean ± SD (*n* = 6). *p <  0.05 (*n* = 6, treated vs. untreated control cells), one-way ANOVA followed by Tukey’s post-hoc tests

Among the cytokines and growth factors affected by BHLHE40-KD in LM cells, the expression level of HBEGF mRNA is positively correlated with the expression level of BHLHE40 mRNA in all four major subtypes of breast tumors in the TCGA database (Figs. 4 and 5 and Table 1). HBEGF is a heparin-binding epidermal growth factor (EGF)-like growth factor that promotes cell proliferation and invasion through EGF receptor (EGFR) activation [35]. To examine the molecular mechanism underlying BHLHE40-mediated HBEGF transcription, we performed ChIP analysis. BHLHE40 binding to the proximal promoter region of HBEGF was not affected by 1%O_2_/LG (data not shown), indicating that HBEGF transcription activation was not caused by increased BHLHE40-DNA binding. However, 1%O_2_/LG treatment reduced binding of HDAC1 and HDAC2 to the HBEGF promoter (Fig. 6a), which is coincident with increased BHLHE40-HDAC1/2 interaction in the soluble cellular fraction, as detected by reciprocal CoIP followed by IB (Fig. 6b). In cells lacking BHLHE40, HDAC1/HDAC2 remained bound to the promoter region of HBEGF after 1%O_2_/LG treatment (Fig. 6a). This result suggests that BHLHE40 plays a role in facilitating the dissociation of HDAC1/2 from promoters through protein-protein interaction. To examine whether HDAC1/2-DNA binding plays a key role in suppressing transcription of BHLHE40 target genes, we examined the effect of HDAC inhibitors on the mRNA expression of HBEGF, CTGF, and VEGFC. As shown in Fig. 6c, both HDAC2-specific (BRD6688, 10 μM) and pan-HDAC inhibitor (TSA, 2 μM) increased the expression of BHLHE40 target genes in MDA-MB-231 EV and BHLHE40-KD cells, supporting a role for HDAC1/2 in suppressing transcription of BHLHE40 target genes. Taken together, these observations suggest that sequestering HDAC1/2 from DNA binding contributes to BHLHE40-mediated transcription activation.

### HBEGF acts to promote cell survival and migration

**Fig. 7:**
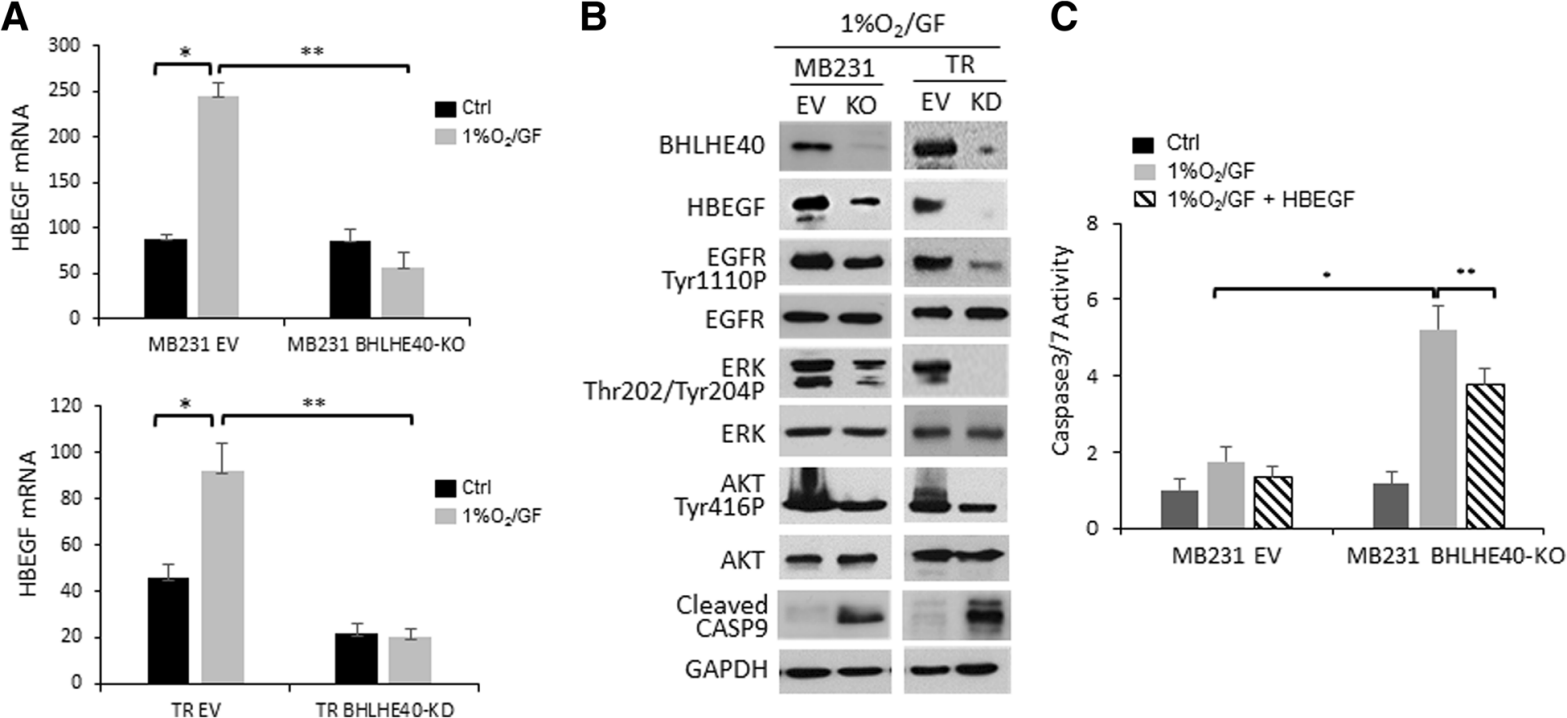
BHLHE40 depletion reduced phosphorylation of epidermal growth factor receptor (EGFR), while it increased Caspase 9 cleavage, in cells exposed to glucose depletion and hypoxia (1%O_2_/GF). **a** BHLHE40-knockout (KO) by CRISPR/Cas9 editing in MDA-MB-231 and BHLHE40-knockdown (KD) by shRNA in tamoxifen resistant (TR) cells diminished HBEGF induction by 1%O_2_/GF (6 h). mRNA expression levels were determined by qPCR, normalized to RPL13A, and presented as mean ± SD (*n* = 6). *p <  0.05 (*n* = 6, 1%O_2_/GF vs. control), **p <  0.05 (*n* = 6, KO vs. EV), one-way ANOVA followed by Tukey’s post-hoc tests. **b** BHLHE40 depletion reduced EGFR activation, as indicated by reduced phosphorylation of EGFR and its downstream targets (ERK and AKT), while increasing apoptosis, as indicated by detection of cleaved caspase 9. Data from three independent immunoblotting analyses are presented. **c** HBEGF peptide (10 μg/ml) reduced apoptosis induced by 1%O_2_/GF (6 h) in MDA-MB-231 BHLHE40-KO cells. Apoptosis was determined by Caspase 3/7 assays. *p <  0.05 (*n* = 6, 1%O_2_/GF vs. control), **p <  0.05 (*n* = 6, HBEGF vs. untreated with HBEGF), one-way ANOVA followed by Tukey’s post-hoc tests

To examine whether BHLHE40-driven HBEGF expression plays a role in EGFR activation to promote cell survival, we examined the phosphorylation status of EGFR and its downstream targets in MDA-MB-231 and TR sublines exposed to 1%O_2_/GF for 6 h, a condition known to induce apoptosis as shown in Fig. 3. Compared with cells with intact BHLHE40 activity, MDA-MB-231-BHLHE40-KO and TR-BHLHE40-KD cells expressed lower levels of HBEGF mRNA and protein, which was coincident with reduced levels of phosphorylation of EGFR, AKT, and ERK, and increased caspase 9 cleavage (Fig. 7a, b). Next, we examined whether active HBEGF peptide could rescue MDA-MB-231-BHLHE40-KO cells from apoptosis. As shown in Fig. 7c, the addition of HBEGF peptide into the culture medium of cells exposed to 1%O_2_/GF significantly reduced activation of caspase 3/7. These observations provide evidence supporting a role of HBEGF in promoting cell survival.

**Fig. 8:**
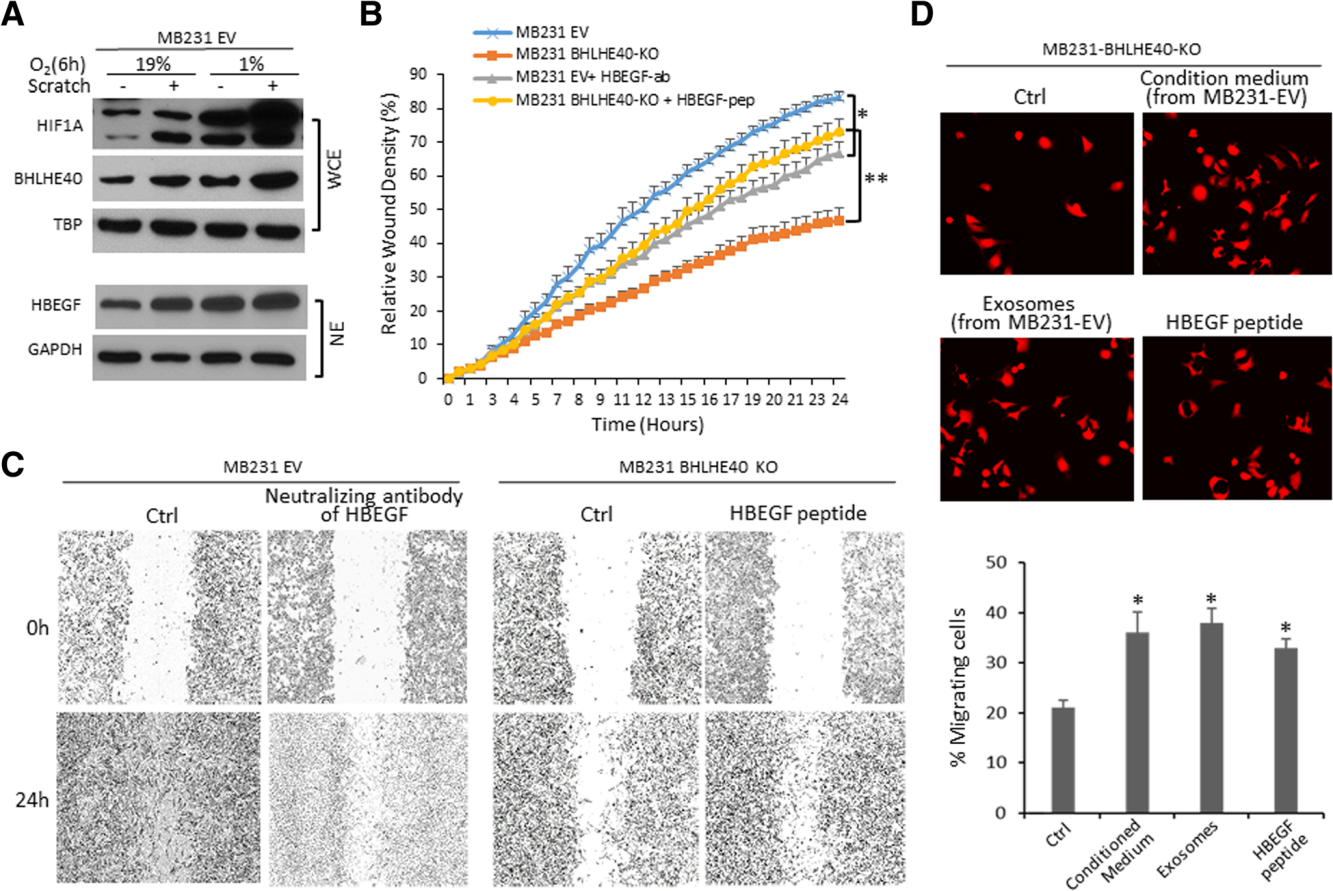
The HIF-BHLHE40-HBEGF axis plays a role in promoting cell migration during wound healing. **a** Monolayer scratch increased protein levels of HIF1A, BHLHE40, and HBEGF in MDA-MB-231 empty vector (EV) cells. Intensive scratch wounds were generated using EMD Millipore’s Cell Comb scratch assay kit and immunoblotting was performed 6 h after cells cultured under normoxia (19% O_2_) or hypoxia (1% O_2_). **b** BHLHE40-knockout (KO) by CRISPR/Cas9 editing reduced the migratory activity of MDA-MB-231 cells, which was restored by the addition of HBEGF peptide into the culture medium. In contrast, a HBEGF-neutralizing antibody reduced the migratory activity of MDA-MB-231-EV cells. Real-time assessment of migratory activity after wound scratch was performed using the IncuCyte ZOOM-ImageLock plate system. *p <  0.05 (*n* = 6, time points 6–24 h, HBEGF vs. untreated), **p <  0.05 (*n* = 6, time points 6–24 h, anti-HBEGF vs. untreated), one-way ANOVA followed by Tukey’s post-hoc tests. Representative data from two independent experiments with six replicates are presented. **c** Images of representative wound fields at 0 and 24 h after wound scratch as described in **b**. **d** Conditioned medium or purified exosomes from MDA-MB-231-EV cells (24 h after wound scratch) increased migratory activities of MDA-MB-231 BHLHE40-KO cells, as determined by the transwell migration assays. The migrated cells in six fields were imaged and counted under fluorescent microscopy. The results are presented as: percent migration = mean number of cells migrating through the uncoated transwells × 100/mean number of seeded cells; *p <  0.05 (*n* = 12, vs. untreated control), one-way ANOVA followed by Tukey’s post-hoc tests

Monolayer scratch was found to induce the expression of HIF1A, BHLHE40, and HBEGF in MDA-MB-231-EV cells (Fig. 8a), implicating a role for the HIF1A-BHLHE40-HBEGF axis in cell migration during wound healing. Using the IncuCyte ZOOM-ImageLock plate system, we demonstrated that BHLHE40-KO substantially diminished the ability of MDA-MB-231 cells to close the wound gaps, which was restored by the addition of HBEGF peptide (Fig. 8b, c). In contrast, a HBEGF-neutralizing antibody [36] inhibited wound healing of LM-EV cells (Fig. 8b, c). To confirm that exosomic HBEGF plays a key role in promoting cell migration, we examined the migratory activities of MDA-MB-231 BHLHE40-KO cells in the presence of conditioned medium or purified exosomes which were collected from the MDA-MB-231 EV cells at 24 h after extensive wound scratch. The transwell migration assay showed that both conditioned medium and purified exosomes from the wounded EV cells increased the migratory activity of BHLHE40-KO cells (Fig. 8d). Together, these observations suggest that HBEGF act downstream of BHLHE40 to promote cell migration.

**Fig. 9:**
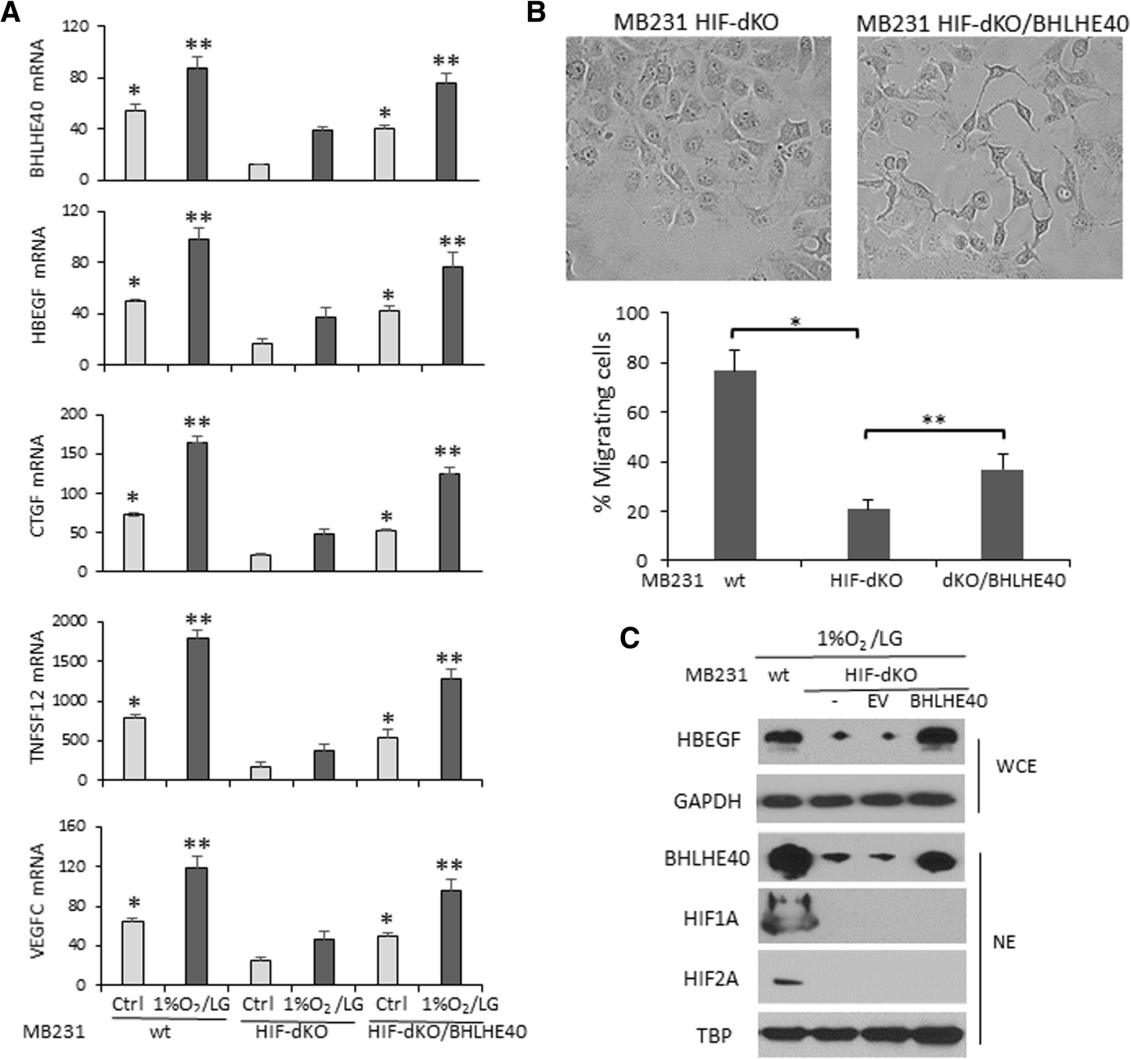
Effect of BHLHE40 overexpression on molecular and phenotypic changes caused by HIF1A/EPAS1 double knockout (HIF-dKO) in MDA-MB-231 cells. **a** BHLHE40 overexpression restored baseline and hypoxia/low glucose (1%O_2_/LG (6 h))-induced expression of HBEGF, CTGF, TNFSF12, and VEGFC in HIF-dKO cells. The expression levels of mRNA were determined by qPCR, normalized to RPL13A and presented as mean ± SD (*n* = 6). *p <  0.05 (*n* = 6, vs. untreated HIF-dKO), **p <  0.05 (*n* = 6, vs. HIF-dKO exposed to 1%O_2_/LG for 6 h), one-way ANOVA followed by Tukey’s post-hoc tests. **b** BHLHE40 overexpression decreased cell-cell contact (as shown by the cell images) and increased migratory activity of HIF-dKO cells exposed to 1%O_2_/LG (24 h). Migratory activity was determined by transwell assays and presented as mean percentage of migrating cells ± SD (*n* = 6). *p <  0.05 (*n* = 6, HIF-dKO vs. control wild-type cells), **p <  0.05 (*n* = 6, HIF-dKO/BHLHE40 overexpression vs. HIF-dKO), one-way ANOVA followed by Tukey’s post-hoc tests. **c** BHLHE40 overexpression restored expression of HBEGF proteins in HIF-dKO cells exposed to 1%O_2_/GF (6 h). Proteins were detected by immunoblotting. GAPDH and TBP were used as loading control for whole cell extract (WCE) or nuclear extract (NE), respectively

To confirm that BHLHE40 and HBEGF are key downstream effectors of HIFs in promoting cell migration, we examined the effect of BHLHE40 overexpression on a MDA-MB-231 subline (HIF-dKO) in which both HIF isoforms (HIF1A and EPAS1) were knocked out by using the CRISPR/Cas9 editing system. Although the HIF1A mRNA expression level is approximately sixfold higher than EPAS1 mRNA in MDA-MB-231 cells according to reported RNAseq data (GSE73526), compensatory activation of EPAS1 could compromise the effect of HIF1A knockout. Therefore, we used HIF-dKO cells to examine whether BHLHE40 overexpression can rescue molecular and phenotypic changes caused by complete elimination of HIF activities. Gene expression analysis by qPCR showed that HIF-dKO reduced baseline and 1%O_2_/LG-induced expression of BHLHE40, HBEGF, CTGF, and VEGFC mRNA, which was restored by BHLHE40 overexpression (Fig. 9a). In addition, BHLHE40 overexpression reduced cell-cell contact, as shown by cell imaging, and increased the migratory activity of HIF-dKO cells, as determined by transwell assays (Fig. 9b). Immunoblotting analysis confirmed that BHLHE40 overexpression restored expression levels of HBEGF protein in HIF-dKO cells exposed to 1%O_2_/LG (Fig. 9c). Together, these observations support the notion that BHLHE40 and HBEGF act as key downstream effectors of HIFs to promote cell migration.

### High expression of BHLHE40 and HBEGF is associated with poor prognosis of breast cancer

**Fig. 10:**
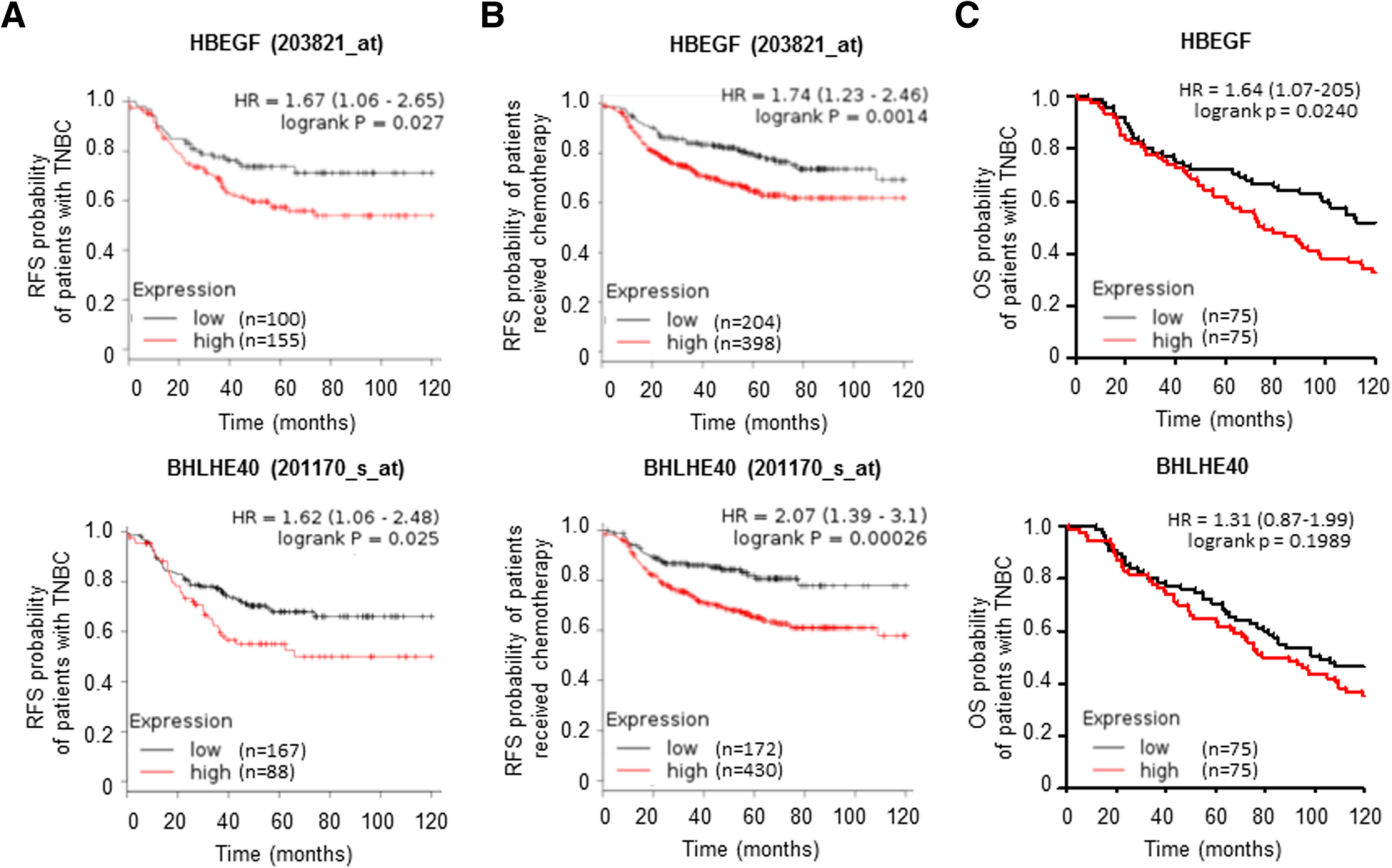
High expression of BHLHE40 and HBEGF is associated with poor prognosis of breast cancer. **a** High expression of BHLHE40 and HBEGF is associated with short interval of relapse-free survival (RFS) of patients diagnosed with triple-negative breast cancer (TNBC; *n* = 255). The gene expression data and patient information were obtained from the Kaplan-Meier plotter database. **b** High expression of BHLHE40 and HBEGF is associated with a short interval of RFS among patients treated with chemotherapy (*n* = 602). The gene expression data and patient information were obtained from the Kaplan-Meier plotter database. **c** High expression of HBEGF is associated with a short interval of overall survival (OS) of patients diagnosed with TNBC (*n* = 150). The gene expression data and patient information were obtained from the METABRIC database in the cBioPortal for Cancer Genomics

Having established a role of the BHLHE40-HBEGF axis in enhancing cell survival and migration, we sought to examine the association of BHLHE40 and HBEGF with clinical characteristics of breast tumors using the gene expression data in the Kaplan-Meier plotter database, which contains the Affymetrix microarray expression data of 2178 breast cancer patients [37]. We found that high expression of BHLHE40 or HBEGF is significantly associated with shorter interval of relapse-free survival (RFS) among patients diagnosed with triple-negative breast cancer (TNBC; *n* = 255) and patients treated with chemotherapy (*n* = 602) (Fig. 10). However, BHLHE40 and HBEGF are not poor prognostic markers for patients with estrogen receptor-positive tumors or patients treated with endocrine therapy. In addition, we analyzed the association of BHLHE40 and HBEGF with overall survival (OS) of TNBC using the METABRIC dataset in the cBioPortal for Cancer Genomics. High HBEGF expression was found to be associated with a short interval of OS (Fig. 10c). Although TNBC with higher expression of BHLHE40 tends to have a shorter interval of OS, this correlation did not reach statistical significance (Fig. 10c). These findings suggest that activation of the BHLHE40-HBEGF pathway contributes to aggressive behaviors of TNBC and chemoresistance.

## Discussion

Breast cancer metastasis is the major cause of death in breast cancer patients. Adaptation to hypoxia is a driving force of metastatic progression and drug resistance [3]. Proteins secreted by tumor cells under hypoxia promote metastasis by altering tumor cell behaviors and modifying the tumor microenvironment [2]. Therefore, the regulation of hypoxia-driven protein secretion is currently under intense investigation. In this study, we report a novel role of BHLHE40, a transcription factor directly targeted by HIF1A, in regulating exosomic release of HBEGF. Our results suggest that the HIF-BHLHE40-HBEGF axis constitutes an important signaling mechanism to promote metastasis of breast tumors.

Exosomes are 40- to 100-nm vesicles that originate from the endocytic compartment. Exosomes contain a wide range of proteins, lipids, mRNAs, and microRNAs (miRNAs) that reflect the molecular contents of the parental cells [32]. Compared with normal cells, cancer cells exhibit higher activity of exosome secretion, which is further augmented by stress conditions including TP53 activation, alteration of intracellular calcium levels, senescence, hypoxia, and acidosis [38]. Exosomes released by tumor cells have been reported to contain cytokines and growth factors that promote metastasis and chemoresistance [38–40]. However, the precise molecular mechanism governing the release of exosomes remains elusive. This study suggests that BHLHE40 acts as a key downstream effector of HIFs to activate transcription and subsequent exosome secretion of a set of cytokines and growth factors.

BHLHE40 was previously described as a transcriptional repressor that binds to the class B E-box (CACGTG) and recruits HDAC1 and HDAC2 to block transcription [41]. BHLHE40 activation has been linked to cell cycle arrest, senescence, differentiation, and apoptosis [42–44]. On the other hand, emerging evidence supports a role for BHLHE40 in transcription activation and promoting cell survival. For instance, BHLHE40 was reported to activate transcription of pro-survival factors in tumor cells, including BIRC5 and DeltaNp63 [45, 46]. In addition, BHLHE40 was reported to activate the transcription of a panel of cytokines required for activation of murine CD4^+^ T cells [47, 48]. Which factors determine the selectivity of BHLHE40 to suppress or activate transcription remains undefined. The BHLHE40-mediated transcription activation of DeltaNp63 was shown to depend on its direct interaction with HDAC2 [45]. In agreement with this observation, our results suggest that BHLHE40 activates HBEGF transcription by sequestering HDAC1/2 from DNA binding. It remains to be determined whether interfering with HDAC1/2-DNA binding is a general mechanism responsible for BHLHE40-mediated transcription activation.

Elevated EGFR activation is known to promote survival, proliferation, and invasion of tumor cells under hypoxia, and multiple mechanisms have been linked to hypoxia-induced EGFR activation [49]. For example, EPAS1 activation by hypoxia was shown to increase EGFR mRNA translation [50]. Hypoxia-mediated activation of metalloproteases (e.g., ADAM12 and ADAM17) was reported to activate EGFR by increasing ectodomain shedding of HBEGF [51, 52]. Our study provides a novel aspect of EGFR activation through the BHLHE40-HBEGF axis. In addition to autocrine or paracrine effects within tumor cells, exosomic release of HBEGF might exert paracrine effects to remodel tumor stroma or endocrine effects to prime distant metastatic niches [53].

In conclusion, this study provides evidence supporting an essential role of BHLHE40 in exosomic release of HBEGF, a critical pro-survival and pro-metastasis factor. The clinical relevance of our findings is evidenced by the fact that the elevated expression of BHLHE40 and HBEGF in breast tumors is associated with poor prognosis of patients with TNBC and chemoresistance. Therapeutic intervention targeting the BHLHE40-HBEGF axis may represent an effective approach to combat hypoxia-driven drug resistance and metastasis.

## Conclusion

Hypoxia-induced activation of BHLHE40 plays a key role in promoting cell survival and metastasis by modulating exosomic secretion of HBEGF.

## Abbreviations

BHLHE40: Basic helix-loop-helix family member E40
ChIP: Chromatin immunoprecipitation
CoIP: Co-immunoprecipitation
EGF: Epidermal growth factor
EGFR: Epidermal growth factor receptor
EV: Empty vectors
FR: Fulvestrant-resistant
GF: Glucose depletion
HBEGF: Heparin-binding epidermal growth factor
HDAC: Histone deacetylases
HIF: Hypoxia inducible factor
HIF-dKO: HIF1A and EPAS1 double knockout
IB: Immunoblotting
KD: Knockdown
KO: Knockout
LG: Low glucose
LM: Lung metastatic cell line derived from MDA-MB-231
NSG: NOD.Cg-Prkdcscid Il2rgtm1Wjl/SzJ
OS: Overall survival
qPCR: Quantitative polymerase chain reaction
RFS: Relapse-free survival
shRNA: Short hairpin RNA
TCGA: The Cancer Genome Atlas
TNBC: Triple-negative breast cancer
TR: Tamoxifen-resistant
